# *Salmonella enterica* Serovar Typhi: An Unusual Cause of Infective Endocarditis

**DOI:** 10.3390/tropicalmed3010035

**Published:** 2018-03-16

**Authors:** Christopher Robson, Matthew V. N. O’Sullivan, Shobini Sivagnanam

**Affiliations:** 1 Department of Infectious Diseases, Blacktown Hospital, Blacktown, NSW 2148, Australia; Shobini.Sivagnanam@health.nsw.gov.au; 2 Centre for Infectious Diseases and Microbiology, Westmead Hospital, Westmead, NSW 2145, Australia; Matthew.OSullivan@health.nsw.gov.au; 3 Marie Bashir Institute for Infectious Diseases and Biosecurity, University of Sydney, Westmead, NSW 2145, Australia

**Keywords:** *Salmonella* Typhi, infective endocarditis, relapse, mitral valve

## Abstract

While typhoid fever is a common infection, *Salmonella enterica* serovar Typhi is a rare cause of endocarditis. We describe the case of a 20-year-old male who was treated for a primary episode of microbiologically-confirmed typhoid fever. He presented six weeks post-discharge with fever and lethargy. *S.* Typhi was again identified in blood cultures, and echocardiography identified a mitral valve lesion. Our case suggests that a relapse of typhoid should prompt further investigation for a deep-seated infection, including consideration of echocardiographic evaluation to rule out infective endocarditis.

## 1. Case Report

We describe the case of a 20-year-old Filipino male who presented to our Australian metropolitan hospital with a history of fever and diarrhoea. He had no significant past medical history and was not on any regular medications. He was born in the Philippines and migrated to Australia in 2008, where he was working as a chef.

He developed symptoms of fevers up to 40 °C, mild headache and watery diarrhea, two weeks after returning from a three-week trip to the Philippines. During the trip he had no sick contacts and no significant exposures to animals or fresh water. He did not receive vaccination for typhoid prior to travel. He was prescribed amoxicillin along with paracetamol and metoclopramide by his local doctor without symptomatic improvement. Hence, he presented to the emergency department seven days after symptom onset.

On presentation, he was febrile at 39.5 °C, with heart rate 105/min, and was hypotensive (80/60 mmHg); however, his blood pressure improved with intravenous fluid resuscitation. His abdomen was soft with mild tenderness in the right upper quadrant. Respiratory and cardiovascular examinations were unremarkable and there were no rashes. Initial investigations revealed haemoglobin (Hb) 141 g/L, white cell count (WCC) 3.9 × 10^9^/L and platelets 101 × 10^9^/L; creatinine 90 μmol/L, elevated C-reactive protein of 218 mg/L, and deranged liver function tests (alanine aminotransferase [ALT] 421 U/L, aspartate aminotransferase [AST] 743 U/L, gamma-glutamyl transferase [GGT] 171 U/L, alkaline phosphatase [ALP] 175 U/L, bilirubin 14 μmol/L). Chest X-ray and urinalysis were unremarkable; serological tests revealed negative Epstein–Barr virus IgM, positive cytomegalovirus IgG but IgM negative, and negative hepatitis B surface antigen, hepatitis C antibody and hepatitis A IgM. An ultrasound of the abdomen identified mild hepatosplenomegaly with no focal lesions. He was commenced on empiric intravenous ceftriaxone 2 g/day.

Blood cultures taken on presentation grew *Salmonella enterica* serovar Typhi. Susceptibility testing, performed using the Phoenix automated broth microdilution system (BD Diagnostic Systems, Sparks, MD, USA), indicated susceptibility to ampicillin (minimum inhibitory concentration [MIC] <4 µg/mL), ceftriaxone (MIC <0.5 µg/mL) and cotrimoxazole (MIC <1/19 µg/mL), using Clinical and Laboratory Standards Institute (CLSI) breakpoints. Ciprofloxacin susceptibility was confirmed using nalidixic acid disc diffusion testing according to CLSI Guidelines (zone size 25 mm, using a 30 μg nalidixic acid disc). Azithromycin susceptibility testing, performed using E-test (bioMérieux, Baulkham Hills, Australia), indicated susceptibility with an MIC of <4 µg/mL. Two further blood cultures taken on days one and two of admission were also positive for *S.* Typhi but were subsequently negative after 24 h of therapy. A stool sample showed no leucocytes or erythrocytes and was positive for *Salmonella* species by polymerase chain reaction (PCR), despite not growing on culture.

Intermittent fevers were present for five days after commencing therapy; however, his overall condition improved substantially, with resolution of diarrhoea and a reduction in inflammatory markers and liver function tests. He received eight days of ceftriaxone before being discharged with a further seven-day course of oral ciprofloxacin 500 mg twice daily. He had complete resolution of his symptoms at the conclusion of this course.

Six weeks after discharge, the patient returned to the emergency department with a five-day history of fevers, lethargy and mild headache. He had no abdominal pain or diarrhoea and no respiratory or urinary symptoms. Examination of his cardiovascular system demonstrated dual heart sounds with no murmurs; respiratory and gastrointestinal system examinations were also unremarkable. There were no peripheral stigmata of infective endocarditis. Initial investigations revealed mild pancytopenia (Hb 123 g/L, WCC 2.3 × 10^9^/L: neutrophils 1.6 × 10^9^/L lymphocytes 0.7 × 10^9^/L, platelets: 127 × 10^9^/L) and creatinine 92 µmol/L, along with elevated C-reactive protein of 110 mg/L and mildly deranged liver function tests (ALT 117 U/L, AST 194 U/L, GGT 30 U/L, ALP 67 U/L, bilirubin 10 µmol/L). A single set of blood cultures taken grew *S.* Typhi, and the pathogen was also demonstrated on faecal culture and PCR. He was commenced on intravenous ceftriaxone 2 g daily.

Multiple investigations were undertaken to identify a possible occult infective focus. Computed tomography scans of brain, chest, abdomen and pelvis were normal, as was a nuclear medicine bone scan. An ultrasound of the abdomen was unremarkable, with a normal appearance of the gallbladder with no cholelithiasis. A transthoracic echocardiogram identified a small, mobile vegetation on the anterior leaflet of the mitral valve. The remaining valves were structurally normal; there was no evidence of abscess formation and no features to suggest myocarditis. The 4 mm mitral valve vegetation was again demonstrated on transoesophageal echocardiography and a diagnosis of *S.* Typhi endocarditis was thus reached.

He was treated with a six-week course of intravenous ceftriaxone 2 g/day, followed by six weeks of oral ciprofloxacin 500 mg twice daily. His blood cultures cleared rapidly within 24 h of treatment commencement, and his symptoms resolved within the first week. Monitoring of inflammatory markers showed normalisation of the C-reactive protein and full blood count parameters. A repeat transoesophageal echocardiogram at the end of treatment demonstrated a 2 mm calcified mass on the anterior leaflet of the mitral valve, consistent with a healed vegetation. Three sets of follow-up blood cultures taken one month following treatment completion remained culture-negative. He did not have further stool examination after completion of therapy. At three months of follow-up, he remained systemically well.

## 2. Discussion

Typhoid fever is an infection caused by the Gram-negative bacillus *S.* Typhi or *S.* Paratyphi A, with most cases due to *S.* Typhi [1,2]. Each year, approximately 22 million new cases of typhoid occur globally, resulting in more than 200,000 deaths [3]. Advances in public health and hygiene measures have resulted in virtual elimination of typhoid fever in developed countries; however, imported cases from endemic countries remain frequent due to international travel.

The disease is characterised by fever, constipation or diarrhoea, abdominal pain, headache and rash [2]. The mainstay of diagnosis is a positive blood culture, although blood cultures are positive in only 40–50% of cases [4,5]. Stool and urine culture sensitivities are much lower [6]. Bone marrow cultures are positive in 80–90% of patients [4,5], although this method of diagnosis is not usually practical or necessary.

Traditionally, first-line agents used for treatment have included chloramphenicol, ampicillin and trimethoprim-sulfamethoxazole. However, increasing rates of resistance to these agents have necessitated the use of fluoroquinolones and third-generation cephalosporins as first-line therapy. In recent years, fluoroquinolone-resistant strains have also emerged, with rates of resistance of up to 13% reported in some regions [7]. This has led to increased usage of azithromycin as an alternative oral therapy. Recommended treatment duration depends on the antibiotic used, but is generally 7–14 days [8].

In spite of appropriate antibiotic therapy, 5–10% of patients experience relapse of the disease after apparent symptom resolution [9]. The factors that mediate disease relapse are poorly understood, but there is evidence in animal models that typhoid bacteria persist in mesenteric lymph nodes following treatment, and that this reservoir may be responsible for repeat dissemination of the pathogen [10].

Approximately 1–6% of patients with typhoid fever go on to develop chronic asymptomatic carriage of the organism. This is defined as excretion of *S.* Typhi in stool or urine more than 12 months following acute infection. There is evidence to suggest that the organism establishes a biofilm on gallstones, and cholelithiasis is a known risk factor for developing chronic carriage [11]. Chronic carriers allow the ongoing transmission of typhoid, with no other known vectors for the disease.

Cardiac involvement in typhoid is well documented, with guidelines recommending routine evaluation of all patients with *Salmonella* species bacteraemia for cardiovascular complications [12]. Up to 4% of patients with typhoid develop myocarditis or pericarditis [13]; endocarditis accounts for 0.01–2.9% of cases amongst large case series [14]. However, there are no published epidemiological data specific to typhoid-associated endocarditis, with only case reports in the literature. Table 1 outlines all cases of *S.* Typhi endocarditis reported in the literature over the past 20 years.

Similar to our case, affected patients were generally less than 50 years of age. The aortic and mitral valves were the most commonly involved; however, unlike in our patient, who had a structurally normal heart, the majority of cases occurred in patients with pre-existing valvular abnormalities. Interestingly, most reported cases had endocarditis diagnosed at initial presentation, whereas our patient was only diagnosed following relapse of symptoms. Our patient had a structurally normal heart, and did not have any clinical or examination features that pointed towards infective endocarditis on his initial or subsequent presentations. It is unclear whether he had an undiagnosed and hence under-treated *Salmonella* endocarditis initially that resulted in relapse of the infection, or whether his relapse was due to recurrent bacteraemia and valvular seeding from persistence of the organism in the gallbladder or reticuloendothelial system.

There are no standardised guidelines on the recommended antibiotic regimen or treatment duration for *Salmonella* endocarditis. Treatment regimens in published cases generally consisted of a third-generation cephalosporin with or without an aminoglycoside for four to six weeks. None of the published cases required surgical intervention, and there were no documented instances of treatment failure. Our patient was treated with an initial six-week course of intravenous ceftriaxone for infective endocarditis. Given his occupation in the food industry, he received a subsequent six-week course of oral ciprofloxacin to eliminate chronic carriage of *Salmonella*.

Our case demonstrates an unusual presentation of typhoid relapse and raises the question of how to appropriately investigate and follow up with these patients. As relapses can occur weeks after apparently ‘successful’ treatment, it is imperative that these patients be followed up for a prolonged period of time. In addition to checking for antimicrobial resistance, we suggest that relapse of symptoms should prompt further investigation for a deep-seated infection, including clinical assessment for evidence of infective endocarditis, with a low threshold for performing echocardiography, even in patients with no pre-existing valvular abnormalities.

## Figures and Tables

**Table 1:** Cases of *Salmonella enteric* serovar Typhi infective endocarditis in the English literature 1997–2017.

Publication	Age/Sex	Cardiac Abnormality	Affected Valve	Treatment	Outcome
Khan et al. 2003 [15]	25/M	Bicuspid AV	Aortic	CRO + AMK 2 weeks → CRO 2 weeks	Recovery
Wani et al. 2004 [16]	45/F	Nil	Mitral	AMP + GEN → CRO + AMK	Recovery
Khanal et al. 2004 [17]	27/F	RHD: MS + MR	Mitral	CIP + GEN	Recovery
Ozer et al. 2009 [18]	27/F	RHD: MS + AR	Aortic	CRO 6 weeks	Recovery
Khan et al. 2011 [19]	21/M	MV repair	Mitral	CRO + GEN 2 weeks → CRO 2 weeks	Recovery

M: Male, F: Female, AV: aortic valve, RHD: rheumatic heart disease, MS: mitral stenosis, MR: mitral regurgitation, AR: aortic regurgitation, MV: mitral valve, CRO: ceftriaxone, AMK: amikacin, AMP: ampicillin, GEN: gentamicin, CIP: ciprofloxacin.
